# National trends and survival outcomes associated with non-guideline-concordant treatment of inflammatory breast cancer

**DOI:** 10.1007/s10549-025-07669-8

**Published:** 2025-03-18

**Authors:** Elsa A. Kronen, Nikhil L. Chervu, Ayesha P. Ng, Hanjoo Lee, Peyman Benharash, Carlie K. Thompson

**Affiliations:** 1 Cardiovascular Outcomes Research Laboratories, David Geffen School of Medicine at UCLA, Los Angeles, CA USA; 2 Department of Surgery, David Geffen School of Medicine at UCLA, Los Angeles, CA USA; 3 Department of Surgery, Harbor-UCLA Medical Center, Torrance, CA USA; 4 UCLA Revlon Breast Center, 100 Medical Plaza, Suite 310, Los Angeles, CA 90095 USA

**Keywords:** Inflammatory breast cancer, National Comprehensive Cancer Network, Trimodal therapy, Survival, Mastectomy, Axillary lymphadenectomy

## Abstract

**Purpose:**

National Comprehensive Cancer Network (NCCN) guidelines for inflammatory breast cancer (IBC) recommend trimodal therapy: neoadjuvant chemotherapy, modified radical mastectomy (MRM), and adjuvant radiation therapy. Historically, a minority received NCCN-guideline-concordant trimodal therapy (GCT). We explored factors associated with non-concordance, types of non-concordance, and the association of GCT with survival.

**Methods:**

The National Cancer Database (NCDB) was analyzed for patients with non-metastatic IBC who underwent surgery from 2006 to 2019. Multivariate logistic regression identified factors associated with GCT. Cox proportional hazard models assessed the impact of GCT, and components of non-concordance, on mortality.

**Results:**

Of 13,733 patients, 47.6% received GCT. Of non-GCT patients, 39.7% had mixed non-concordance, 25.5% exclusive chemotherapy non-concordance, 24.0% exclusive radiation non-concordance, and 10.8% exclusive surgical non-concordance. A higher burden of comorbidities, node-negative disease, and positive human epidermal growth factor receptor 2 (HER2) and hormone receptor status were associated with reduced odds of receiving GCT. GCT was associated with reduced 3-(hazard ratio [HR] = 0.83, 95% CI 0.73–0.95) and 5-year (HR = 0.83, 95% CI 0.76–0.91) mortality. Chemotherapy non-concordance and mixed non-concordance were associated with higher three-(HR = 1.28, 95% CI 1.07–1.53; HR = 1.20, 95% CI 1.01–1.43 respectively) and 5-year (HR = 1.42, 95% CI 1.26–1.60; HR = 1.17, 95% CI 1.03–1.33) mortality. Radiation non-concordance was associated with increased hazard of 1-year mortality (HR = 3.03, 95% CI 1.75–5.23). Exclusive surgical non-concordance was not associated with survival; however, simple mastectomy portended a higher hazard of 5-year mortality (HR = 1.26, 95% CI 1.08–1.46).

**Conclusion:**

Despite improved survival, a minority of patients received GCT. Omitting neoadjuvant chemotherapy or adjuvant radiation was associated with reduced survival, whereas surgical non-concordance in patients with concordant chemoradiation did not impact survival. Simple mastectomy was associated with reduced survival, supporting the rationale for axillary lymphadenectomy.

## Introduction

Inflammatory breast cancer (IBC) comprises only 1–5% of all breast cancer cases in the United States but accounts for a disproportionately large number of breast cancer deaths, with half of patients not surviving beyond 5 years after diagnosis [1–3]. In contrast to the treatment of non-IBC, which has evolved toward limiting the extent of surgical, systemic, and radiation therapies when it is safe to do so, optimal treatment for IBC still warrants an aggressive multimodal approach. National Comprehensive Cancer Network (NCCN) guidelines recommend neoadjuvant systemic therapy, followed by modified radical mastectomy (MRM) and adjuvant radiation therapy (“trimodal therapy”), as well as adjuvant endocrine therapy, targeted therapy, and immunotherapy for appropriate IBC patients.

Prior studies [4–6] have noted that a minority of patients with IBC receive care that is fully concordant with NCCN guidelines despite data demonstrating improved 5- and 10-year survival for patients receiving trimodal therapy [7]. Surprisingly, the proportion of patients receiving trimodal therapy peaked in 2004 and has declined since, despite NCCN guidelines remaining largely unchanged since 2000 [7, 8]. Furthermore, studies have shown increasing use of sentinel lymph node biopsy (SLNB) for IBC despite a lack of evidence supporting the safety of less extensive axillary surgery [9]. A contemporary analysis of the patient and center-level factors associated with fully concordant care, and the impact of specific treatment domains—surgical, medical, or radiation—on survival of IBC patients remains lacking.

The present study used a national cohort of patients undergoing surgery for IBC to examine 5-year survival outcomes associated with receipt of guideline-concordant therapy. Furthermore, we examined trends in treatment patterns for IBC over the 14-year study period. We hypothesized that concordance with NCCN guidelines for treatment of IBC has decreased over time, partially driven by a rise in non-concordant surgical treatment such as less extensive axillary surgery, and that non-guideline-concordant therapy would be associated with increased mortality.

## Methods

This was a retrospective cohort analysis using the 2006–2019 Nationwide Cancer Database (NCDB). Jointly managed by the American College of Surgeons Commission on Cancer and the American Cancer Society, the NCDB is the largest nationally representative oncologic database, capturing nearly 70% of all newly diagnosed cancer patients in the United States [10]. The data used for this study were derived from a de-identified NCDB file.

**Fig. 1:**
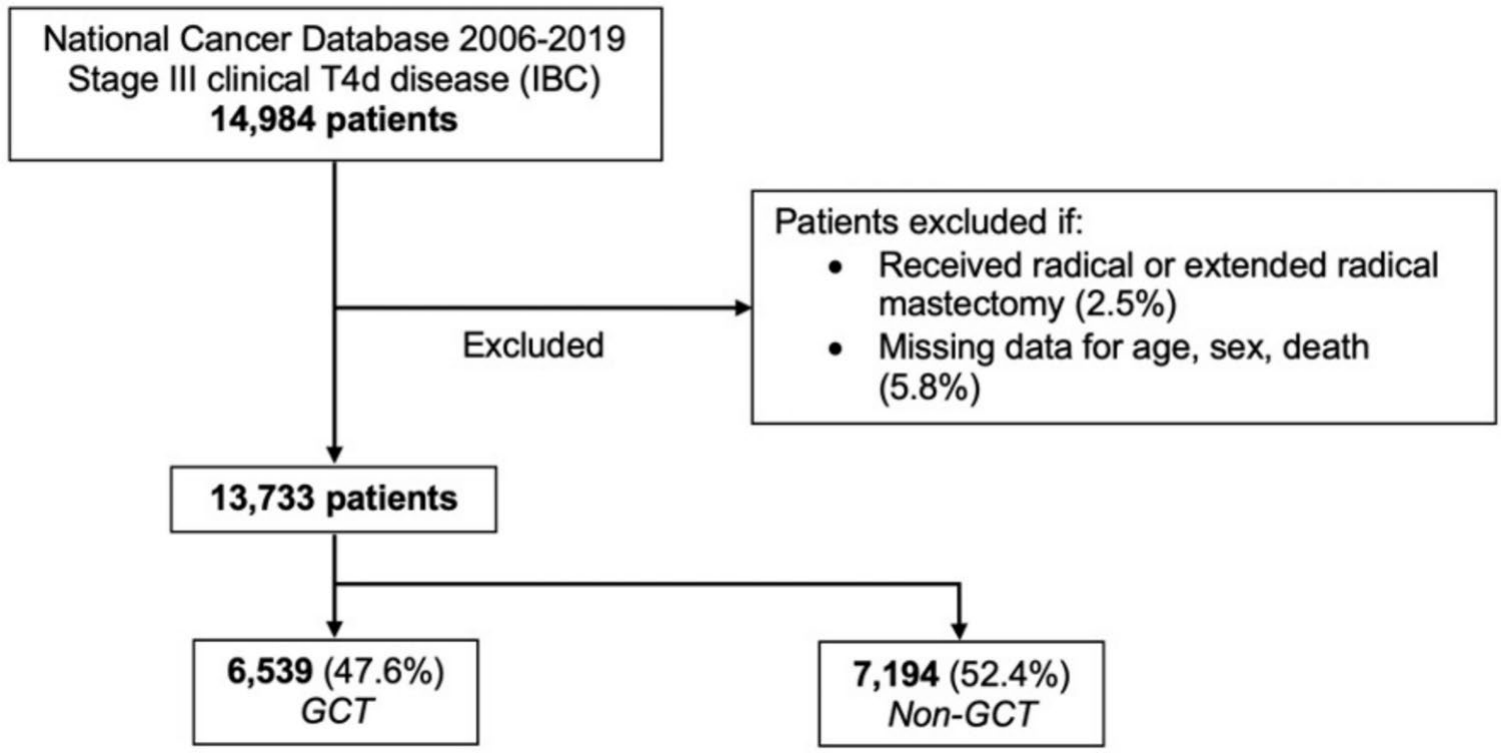
CONSORT diagram with survey-weighted estimates. *IBC* inflammatory breast cancer, *GCT* guideline-concordant therapy

All adult (≥ 18 years) female patients who underwent surgery for IBC were identified using the *International Classification of Diseases for Oncology, Third Edition* codes. Patients who received resection without curative intent were excluded from analysis. The presence of IBC was defined by the American Joint Committee on Cancer as stage T4d without metastatic disease. To account for the multiple revisions of the staging system that occurred during the study period, tumor stage was updated to the 8th edition using the CS_EXTENSION variable, tumor size, and T stage [11, 12]. Records with missing data for age, sex, and death were excluded from analysis (5.8%), as well as patients who underwent more extreme surgery (radical mastectomy and extended radical mastectomy) (2.5%, Fig. 1).

Patient demographic factors including age, race, income, insurance status, geographic location, and education level attained were recorded, with urban/rural status derived using zip-code-level data. Disease factors including clinical T stage, tumor size, tumor grade, nodal status, and receptor status were defined as reported in the NCDB. To quantify the burden of comorbidities, the Charlson-Deyo Index was calculated using validated methodology that sums scores assigned to 17 comorbid conditions as previously described [13]. Modalities and sequence of treatment modalities (type of surgery, chemotherapy, radiation, hormone, and immunotherapy) were tabulated for each patient. In accordance with current NCCN recommendations, guideline-concordant therapy (GCT) was defined by neoadjuvant chemotherapy, MRM or total mastectomy with axillary lymphadenectomy (ALND), and adjuvant radiation, in addition to hormonal therapy for estrogen or progesterone receptor (ER/PR) positive cancers. Immunotherapy use was not used to determine concordance, as human epidermal growth factor receptor 2 (HER2) status was not recorded prior to 2010. We defined MRM as a simple mastectomy with ALND (≥ 6 lymph nodes removed) with or without removal of uninvolved contralateral breast and MRM with or without removal of uninvolved contralateral breast. Non-GCT was categorized into exclusively surgical non-concordance (including lumpectomy and simple mastectomy with SLNB alone), non-concordance due to lack of neoadjuvant chemotherapy or adjuvant radiation therapy, and multifactorial (mixed) non-concordance, including lack of hormonal therapy for ER/PR-positive tumors. Patients receiving concordant treatment comprised the GCT group (others: non-GCT).

Categorical variables were compared using Pearson’s *χ*^2^ test and are reported as group proportions (%). Continuous variables were compared using the Mann–Whitney *U* test and are shown as means with standard deviation (SD). We used Cuzick's nonparametric rank-based test to examine the statistical significance of temporal trends (*nptrend*) [14]. A multivariable logistic regression model was developed to assess risk-adjusted associations between patient, tumor, and hospital factors and the receipt of GCT. Cox proportional hazard models were constructed to assess the impact of GCT on hazard of 3- and 5-year mortality. A log-rank test was utilized to compare survival distributions between cohort subgroups which were graphically depicted using the Kaplan–Meier method. The proportional-hazards assumptions were checked on the basis of Schoenfeld residuals after fitting the model. Regression outputs are reported as adjusted odd ratios (AOR) or hazard ratios (HR) with 95% Confidence Intervals (CI). Statistical significance was set at *α* = 0.05. All data extraction and statistical analyses were performed using STATA 16.1 (StataCorp, College Station, TX). Given that the NCDB is fully de-identified, this study was deemed exempt from full review by the Institutional Review Board at the University of California, Los Angeles.

## Results

### Trend analysis

The annual incidence of cancers classified as IBC remained relatively stable over the study period, from 0.54% in 2006 to 0.40% in 2019. Of 13,733 patients with non-metastatic clinical T4d IBC, 6539 (47.6%) received GCT and 7194 (52.4%) received non-GCT (“GCT” and “non-GCT” cohorts). The proportion of GCT peaked in 2011 and was significantly lower during the more recent half of this study period (51.2% in 2006–2012 vs. 43.9% in 2013–2019, *p* < 0.001).

### Baseline characteristics

Compared to the GCT cohort, non-GCT patients were older (57.7 vs 55.4 years, *p* < 0.001), more frequently publicly insured (44.6 vs 36.2%, *p* < 0.001), in the lowest quartile of educational achievement (2.5 vs 1.7%, *p* = 0.01), and had a higher burden of comorbidities (Charlson ≠ 0: 18.2 vs. 16.6%, *p* < 0.001). Regarding disease characteristics, the non-GCT cohort more frequently had node-negative disease (12.2 vs 7.7%, *p* < 0.001) and were more likely to be HR+/HER2+ (1.2 vs 0.2%, *p* < 0.001).

There were significant differences between cohorts regarding the location of care. GCT was more common in the Midwest (26.0 vs 21.8%, *p* < 0.001) and in integrated health systems (18.8 vs 17.2%, *p* < 0.001), while non-GCT was more common in the South (36.4 vs 32.6%, *p* < 0.001), and in community hospitals (45.4 vs 41.9%, *p* < 0.001) (Table 1).

**Table 1:** Patient, clinical, and hospital characteristics of patients diagnosed with IBC with (GCT) or without (non-GCT) guideline-concordant therapy

	Non-GCT (*n* = 7194)	GCT (*n* = 6539)	*p*-value
*Patient Characteristics*			
Age (years, mean ± SD)	57.7 ± 13.6	55.4 ± 12.1	< 0.001
Race (%)			0.58
White	5725 (79.6)	5235 (80.0)	
Black	1107 (15.4)	1000 (15.3)	
Asian/Pacific Islander	176 (2.5)	163 (2.5)	
Other/Unspecified	186 (2.6)	141 (2.2)	
Income Quartile (%)			0.55
76th–100th	5137 (71.4)	4715 (72.1)	
51st–75th	414 (5.8)	354 (5.4)	
26th–50th	407 (5.7)	359 (5.5)	
0–25th	465 (6.5)	388 (5.9)	
Unspecified	771 (10.7)	723 (11.1)	
Primary Payer (%)			< 0.001
Private	3528 (49.0)	3739 (57.2)	
Medicare	2221 (30.9)	1510 (23.1)	
Medicaid	982 (13.7)	857 (13.1)	
Uninsured	282 (3.9)	265 (4.1)	
Other/Unspecified	181 (2.5)	168 (2.6)	
Education Quartile (%)			0.01
76th–100th	5993 (83.3)	5456 (83.4)	
51st–75th	116 (1.6)	129 (2.0)	
26th–50th	144 (2.0)	121 (1.9)	
0–25th	177 (2.5)	111 (1.7)	
Unspecified	764 (2.5)	722 (11.0)	
Charlson-Deyo Index (%)			< 0.001
0	5888 (81.9)	5450 (83.4)	
1	975 (13.6)	878 (13.4)	
≥ 2	331 (4.6)	211 (3.2)	
*Tumor Characteristics*			
Tumor Size (mm, mean ± SD)	59. ± 77.1	57.1 ± 63.1	0.06
Nodal Status (%)			< 0.001
0	878 (12.2)	504 (7.7)	
1	2599 (36.1)	2429 (37.2)	
2	1432 (19.9)	1682 (25.7)	
3	1287 (17.9)	1421 (21.7)	
X	991 (13.8)	503 (7.7)	
Grade (%)			0.24
Well Differentiated	158 (2.2)	146 (2.2)	
Moderately Differentiated	1641 (22.8)	1503 (23.0)	
Poorly Differentiated	3483 (48.4)	3250 (49.7)	
Unspecified	1912 (26.6)	1640 (25.1)	
Receptor Status (%)			< 0.001
HR+/HER2−	1184 (16.5)	1412 (21.6)	
HR+/HER2+	103 (1.4)	12 (0.2)	
HR−/HER2+	719 (10.0)	1044 (16.0)	
HR−/HER2−	1317 (18.3)	1545 (23.6)	
Unspecified	3871 (53.8)	2526 (38.6)	
*Hospital Characteristics*			
Hospital Region (%)			< 0.001
Northeast	1264 (17.6)	1105 (16.9)	
Midwest	1566 (21.8)	1699 (26.0)	
South	2619 (36.4)	2130 (32.6)	
West	1106 (15.4)	938 (14.3)	
Unspecified	639 (8.9)	667 (10.2)	
Hospital Type (%)			< 0.001
Academic	2053 (28.5)	1901 (29.1)	
Community	3265 (45.4)	2741 (41.9)	
Integrated Network	1237 (17.2)	1230 (18.8)	
Urban Hospital (%)			0.48
Metropolitan	5754 (80.0)	5241 (80.2)	
Urban	1068 (14.9)	955 (14.6)	
Rural	142 (2.0)	150 (2.3)	
Unspecified	230 (3.2)	193 (3.0)	

*SD* standard deviation , *mm* millimeter

### Non-guideline concordant treatment

Among the non-GCT cohort, 25.5% demonstrated exclusively chemotherapy non-concordance, 10.8% exclusively surgical non-concordance, 24.0% exclusively radiation therapy non-concordance, and 39.7% mixed non-concordance (Table 2). In the patients who did not receive guideline-concordant surgical care, 25.8% underwent a lumpectomy, 37.8% underwent mastectomy with SLNB alone, 34.6% underwent mastectomy with no axillary procedure, and 1.7% underwent mastectomy with unknown axillary surgery. Of note, approximately 11% of mastectomy patients in both the non-GCT and GCT cohorts underwent immediate or delayed reconstruction. 46.6% received an autologous reconstruction, 40.0% an implant reconstruction, and 13.4% a combined reconstruction.

**Table 2:** Proportion of patients receiving suboptimal surgery, chemotherapy, radiation, or hormone therapy in the entire cohort

	Non-GCT *N* (%)
Surgical Non-concordance	1856 (13.5)
Lumpectomy	480 (25.9)
SLNB (% of all lumpectomy)	103 (21.5)
ALND	198 (41.3)
No Axillary Procedure	171 (35.6)
Unknown Axillary Procedure	8 (1.7)
Simple Mastectomy	1376 (74.1)
SLNB	702 (51.0)
No Axillary Procedure	642 (46.7)
Unknown Axillary Procedure	32 (2.3)
Chemotherapy Non-concordance	3139 (22.9)
Adjuvant Chemotherapy	2573 (82.0)
No Chemotherapy	566 (18.0)
Radiation Non-concordance	3414 (24.9)
Neoadjuvant Radiation	211 (11.9)
No Radiation	1558 (88.1)
Hormone Therapy Non-concordance	
ER/PR Positive and No Hormone Therapy	1372 (10.0)

### Factors associated with GCT

Upon multivariate analysis, the presence of nodal metastases (N1: AOR 2.27 [95% CI 1.74–2.97], N2: AOR 3.00 [95% CI 2.27–3.96], N3: AOR 2.72 [95% CI 2.06–3.61]; ref: N0) was associated with significantly increased odds of GCT. Positive HER2 and hormone receptor status (HR+/HER2+: AOR 0.11 [95% CI 0.04–0.25]) were associated with reduced odds of receiving GCT (Table 3). Upon multivariate adjustment, a higher burden of comorbidities was persistently associated with reduced odds of receiving GCT (AOR 0.87, 95% CI 0.77–0.99). Additionally, Medicare insurance was associated with reduced odds of receiving GCT (AOR 0.76, 95% CI 0.62–0.94). Of note, after multivariate analysis, age, race, differentiation status, and hospital region no longer demonstrated significant association with GCT (Table 3)*.*

**Table 3:** Factors associated with receipt of guideline-concordant therapy (GCT) after multivariate analysis

	AOR	95% CI	*p*-value
*Patient Characteristics*			
Age (per year)	0.99	0.98–1.00	0.02
Race			
White	REF	–	–
Black	1.03	0.84–1.26	0.78
Asian/Pacific Islander	1.02	0.63–1.65	0.94
Other/Unspecified	0.65	0.33–1.28	0.21
Primary Payer			
Private	REF	–	–
Medicare	0.76	0.62–0.94	0.01
Medicaid	0.90	0.71–1.13	0.36
Uninsured	0.92	0.61–1.37	0.67
Other/Unspecified	0.90	0.51–1.62	0.74
Charlson-Deyo Index	0.87	0.77–0.99	0.04
*Tumor Characteristics*			
Tumor Size (per mm)	1.00	1.00–1.00	0.79
Nodal Status (%)			
0	REF	–	–
1	2.27	1.74–2.97	< 0.001
2	3.00	2.27–3.96	< 0.001
3	2.72	2.06–3.61	< 0.001
X	0.61	0.41–0.92	0.02
Grade (%)			
Well Differentiated	REF	–	–
Moderately Differentiated	1.18	0.77–1.81	0.45
Poorly Differentiated	1.06	0.69–1.62	0.81
Receptor Status (%)			
HR−/HER2−	REF	–	–
HR+/HER2+	0.11	0.04–0.25	< 0.001
HR−/HER2+	1.21	0.99–1.48	0.06
HR+/HER2−	0.87	0.73–1.04	0.2
*Hospital Characteristics*			
Hospital Region (%)			
Northeast	REF	–	–
Midwest	1.15	0.92–1.44	0.22
South	0.88	0.71–1.09	0.24
West	1.02	0.79–1.32	0.87
Hospital Type (%)			
Academic	REF	–	–
Community	0.94	0.79–1.11	0.47
Integrated Network	1.07	0.86–1.32	0.55
Urban Hospital (%)			
Metropolitan	REF	–	–
Urban	0.97	0.79–1.19	0.77
Rural	1.32	0.81–2.12	0.26

*AOR* adjusted odds ratio, *95% CI* confidence interval, *REF* reference

### Outcomes

**Fig. 2:**
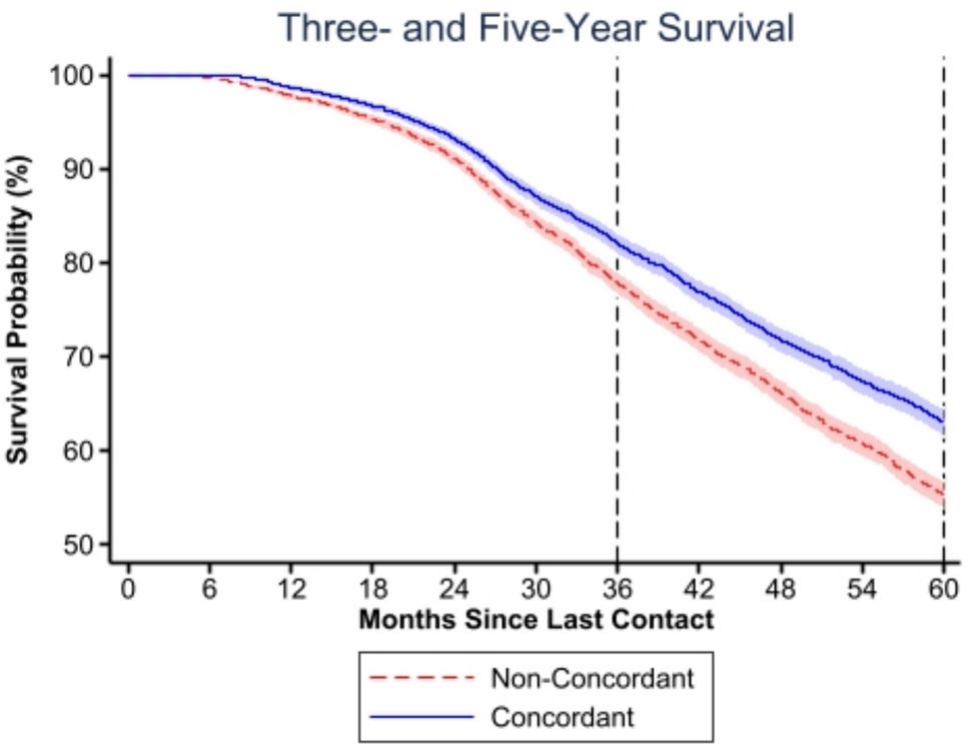
Kaplan–Meier 3- and 5-year survival estimates of patients receiving guideline-concordant vs. non-concordant therapy for inflammatory breast cancer; *p < 0.001 (Proportional-hazards assumption test: 3-year *p* = 0.003; 5-year *p* < 0.001)

On Cox proportional hazard analysis, GCT was associated with significantly reduced hazards of three- (HR 0.83, 95% CI 0.73–0.95) and 5-year mortality (HR 0.83, 95% CI 0.76–0.91) compared to non-GCT. (Fig. 2). Asian/Pacific islander race was associated with greater 1-year mortality (HR 3.38, 95% CI 1.40–8.13), but not 3- and 5-year mortality. Black and “other” race patients demonstrated equivalent 1-year, but increased three- (HR 1.35, 95% CI 1.13–1.61) and 5-year mortality (HR 1.35, 95% CI 1.19–1.53). Having no insurance was associated with an increased hazard of 3-year mortality (HR 1.64, 95% CI 1.22–2.21) and Medicaid was associated with an increased hazard of 5-year mortality (HR 1.24, 95% CI 1.08–1.42).

**Fig. 3:**
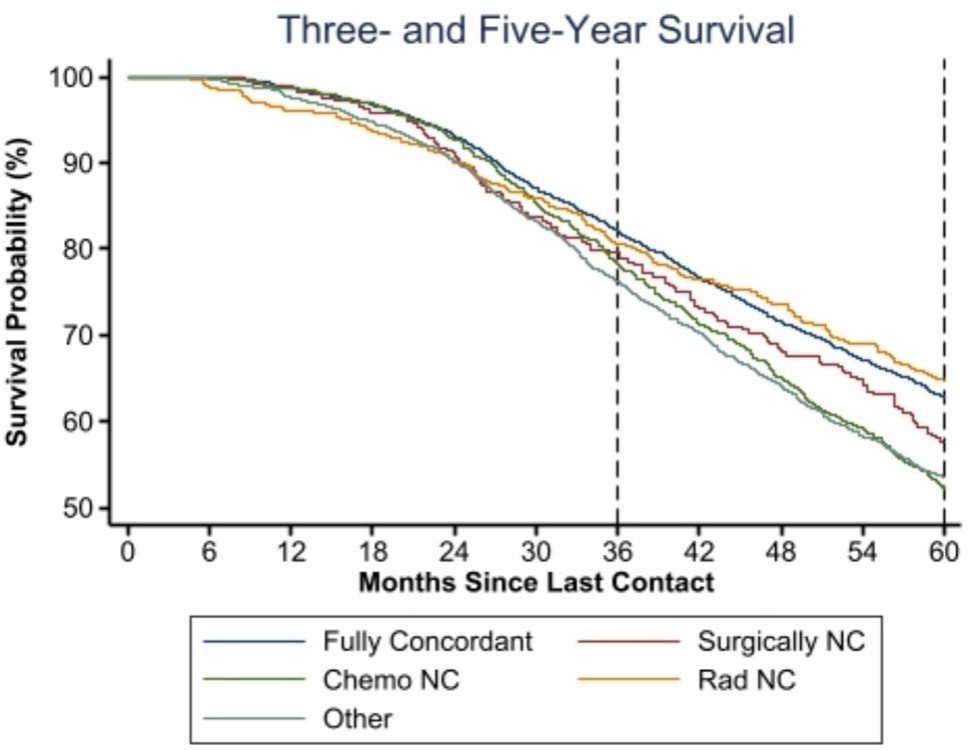
Kaplan–Meier 3- and 5-year survival estimates comparing guideline-concordant vs non-concordant therapy (GCT vs. non-GCT) cohort subgroups: surgical non-concordance (“Surgically NC”), chemotherapy non-concordance (“Chemo NC”), radiation non-concordance (“Rad NC”), or non-concordant for a combination of factors (“Other”); *p < 0.001 (Proportional-hazards assumption test: 3-year *p* = 0.006; 5-year *p* < 0.001)

To evaluate the various types of non-GCT, we analyzed the association of exclusively chemotherapy, surgical, radiation, or other (mixed) non-concordance on survival outcomes. Chemotherapy non-concordance was associated with a 1.3-fold risk of 3-year mortality (HR 1.28, 95% CI 1.07–1.53) and a 1.4-fold risk of 5-year mortality (HR 1.42, 95% CI 1.26–1.60). At 1 year, radiation non-concordance was significantly associated with a threefold increase in hazard of mortality (HR 3.03, 95% CI 1.75–5.23). Mixed non-concordance was also significantly associated with an increase in hazard of three- (HR 1.20, 95% CI 1.01–1.43) and 5-year mortality (HR 1.17, 95% CI 1.03–1.33) (Fig. 3).

Exclusively surgical non-concordance was not significantly associated with odds of 1-, 3-, or 5-year survival. However, we further sought to investigate if specific operations were associated with mortality. Patients who received a simple mastectomy had a significantly higher hazard of 5-year mortality (HR 1.26, 95% CI 1.08–1.46) compared to MRM. Across the entire patient population, including those with mixed non-concordance, lumpectomy did not have a significant association with mortality, although the number of patients who received a lumpectomy was low.

Paradoxically, concordant surgical treatment, regardless of appropriate chemoradiation therapy, was associated with higher one- (HR 1.66, 95% CI 1.05–2.64), three- (HR 1.26, 95% CI 1.10–1.45), and five-year mortality (HR 1.23, 95% CI 1.11–1.35).

## Discussion

Using a nationally representative cohort of patients diagnosed with non-metastatic IBC, we found that over half of patients who started treatment with curative intent did not receive treatment that was in accordance with the NCCN guidelines. Patients with node-negative disease and more medical comorbidities were less likely to receive GCT. Radiation and chemotherapy non-concordance, as well as simple mastectomy, demonstrated a greater hazard of mortality. Several of these findings warrant further discussion.

Guidelines specific for IBC were first established by the NCCN in 2008 [15]. Since 2008 there have been significant changes in systemic therapy regimens, including the addition of targeted therapies and immunotherapies, but the basic premise of trimodal treatment has remained relatively unchanged. Before the introduction of these guidelines, IBC treated with surgery, with or without adjuvant radiation, had a dire survival rate of 5% at 5 years [16]. With the introduction of neoadjuvant chemotherapy, survival rates approximately doubled [17–19]. The use of trimodal therapy is associated with significantly improved overall survival [4, 6–8], and this is corroborated by our findings of a 24% reduction in 3-year mortality for patients who received GCT. Despite this, our study confirmed the findings of others who reported a persistently low rate of adherence to NCCN guidelines and an actual decline in GCT in the U.S. after 2011.

In exploring potential reasons for divergence of treatment from mortality-reducing GCT, our study revealed several factors associated with receiving non-GCT. Upon adjustment, patient-specific disease factors included node-negative disease and triple-positive receptor status, both of which are associated with improved survival outcomes in IBC patients [20]. Healthcare providers may be less eager to refer those with less advanced (i.e., node negative) disease for neoadjuvant chemotherapy. Patients with HER2 positive receptor status tend to have more robust responses to neoadjuvant chemotherapy so the temptation to do less extensive surgery may explain their association with guideline non-concordance. Additionally, in accordance with prior investigations [4, 7], patients with a higher burden of comorbidities were less likely to receive concordant care. This may reflect appropriate triage of patients lacking the physiologic reserve for chemotherapy. However, further investigation may be warranted to ensure that the exclusion of therapy is evidence-based, noting recent studies indicating good tolerance and appropriateness of GCT in the geriatric population [6].

Unadjusted demographic factors associated with non-GCT included public insurance and treatment in the South or at a community hospital, compared to treatment at academic or integrated network centers. These hospital and regional differences are similar to those found in prior investigations and may reflect the challenges in rendering an early diagnosis of the disease [4, 8]. Community hospitals may be disadvantaged both by the rarity of IBC and the lack of advanced technology to facilitate the diagnosis and staging. A study by Jacene et al. [21] demonstrated the potential advantage of FDG/PET-CT, a modality not available in many hospitals, over contrast-enhanced computerized tomography (CT) in staging IBC.

In contrast to prior reports indicating radiotherapy non-concordance as the most common reason for non-GCT [4, 6], our study demonstrated deviations from appropriate neoadjuvant chemotherapy to be the most common reason for non-concordance, with radiation non-concordance closely following. Given that our study confirms the significance of neoadjuvant chemotherapy alone on survival outcomes, it is imperative that IBC patients are expeditiously diagnosed and referred for chemotherapy. Our analysis demonstrated that chemotherapy non-concordance, even with appropriate surgery and radiation therapy, was associated with increased 5-year mortality, emphasizing the importance of chemotherapy as a first-line treatment.

Patients who were non-concordant solely due to lack of radiation therapy experienced a threefold increase in risk of mortality at 1-year but did not exhibit a higher overall 3- or 5-year mortality. This finding suggests the need for a more detailed analysis of this patient population, defining the reasons for the omission of radiation therapy, with the possible consideration for a more tailored approach to radiation therapy in this population.

Our study differs from previous investigations in our detailed definition of surgical concordance. Some prior investigators have considered any surgery, flanked by neoadjuvant chemotherapy and adjuvant radiation therapy, to represent fully concordant trimodal treatment [7]. Others defined "mastectomy," without requiring ALND, as concordant [4]. We believe this granularity is important, as we demonstrated that treatment with simple mastectomy, as compared to MRM, was associated with a 26% increased hazard of 5-year mortality. Although this finding was not replicated in the analysis of patients receiving a lumpectomy, the lack of significance may be due to a small number of patients receiving a lumpectomy. The reduced 5-year survival associated with simple mastectomy appears to be partially mitigated by fully concordant radiation and chemotherapy, as survival in isolated surgical non-concordance was not significantly reduced.

In addition to GCT, we identified demographic factors that were independently associated with mortality. Patients of minority races, including Asian/Pacific islander, Black, and "Other" race, were associated with increased hazard of mortality. This corroborates previous studies that have shown that Black and Hispanic women suffer worse survival outcomes than their white counterparts, even after adjusting for socioeconomic status [4, 22–25]. Uniquely, we identified an increased hazard of mortality for Asian/Pacific Islander patients, contrary to a prior study using Surveillance, Epidemiology, and End Results data [26]. Analysis of differing survival in this group is complicated by the aggregation of Asians with Native Hawaiian/Pacific Islanders, as Native Hawaiian/Pacific Islanders are known to suffer generally poorer prognosis [27]. Demographic-related factors, including being uninsured and under Medicaid, were associated with increased hazard of 3- and 5-year mortality, respectively, which correlates with the well-documented knowledge that socioeconomic status is strongly correlated to limited access to care and poorer survival outcomes.

The present study has several important limitations including those inherent to its retrospective study design and use of an administrative database. The NCDB provides median household income data divided into quartiles based on ZIP codes, which prevents us from treating it as a continuous variable. While we categorized income by the year and place of diagnosis, we acknowledge that socioeconomic conditions can vary widely within a single ZIP code. The NCDB does not contain information on patient preference so we cannot determine if concordance was influenced by patient or clinician decisions. Timing of symptom onset or delays in presentation to the hospital are not available in the NCDB and could have influenced both disease prognosis and surgical decision-making. It also cannot be assessed if surgery-specific factors were influenced by intra-operative findings or events. Details of systemic therapy, such as the specific chemotherapy agents used, their doses, and the number of treatments, were not included, which may have influenced patient outcomes.

It should be noted that our study focused on patients who received resection for curative intent. We believe that patients who received palliative surgery would not necessarily be candidates for trimodal therapy and might more appropriately follow NCCN Guidelines for Palliative Care. Additionally, the 2.5% of patients who underwent more extreme surgery were excluded, as we were concerned that this was a proxy for more advanced disease that would mandate deviation from standard trimodal therapy. Additionally, 5.8% of patients were excluded from analysis due to missing data for age, sex, and death. We cannot be certain that these patients with missing data were reflected in the population included for analysis. Lastly, because this is a retrospective study, we cannot draw any causal relationships from these associations. Despite these limitations, we can confidently conclude that IBC remains a particularly aggressive form of breast cancer.

## Conclusions

Despite consistent NCCN recommendations for trimodal therapy, only about half of patients receive GCT, which is associated with improved survival. Modifiable demographic factors associated with receiving GCT include private insurance and treatment at integrated care centers/hospitals. Omission of neoadjuvant chemotherapy or adjuvant radiation was associated with reduced survival, whereas omission of ALND in patients who received concordant chemoradiation therapy was not associated with reduced survival. Our study confirms the rationale for compliance with NCCN recommendations for surgical management of the axilla, in that simple mastectomy was associated with reduced survival. In the current climate of decreasing rates of GCT, our findings underscore the need for an aggressive approach toward IBC, including care coordination and quality improvement efforts to better align treatment with guidelines.

## Data Availability

Due to the sensitive nature of the data, the dataset cannot be shared publicly. Researchers interested in accessing the NCDB for similar analyses can apply for access through the NCDB Participant User File (PUF) application process. Detailed information about the application process, data availability, and conditions of use can be found on the NCDB website: https://www.facs.org/quality-programs/cancer-programs/national-cancer-database/.

## Abbreviations

NCCN: National Comprehensive Cancer Network
NCDB: National Cancer Database
IBC: Inflammatory breast cancer
GCT: Guideline-concordant treatment
MRM: Modified radical mastectomy
SLNB: Sentinel lymph node biopsy
ALND: Axillary lymphadenectomy
ER/PR: Estrogen receptor/progesterone receptor
HER2: Human epidermal growth factor receptor 2
HR: Hazard ratio
AOR: Adjusted odds ratio
SD: Standard deviation
CI: Confidence interval
CT: Computerized tomography
